# Predictors of adherence to antiretroviral therapy among people living with HIV/AIDS in resource-limited setting of southwest ethiopia

**DOI:** 10.1186/1742-6405-7-39

**Published:** 2010-10-30

**Authors:** Ayele Tiyou, Tefera Belachew, Fisehaye Alemseged, Sibhatu Biadgilign

**Affiliations:** 1 Department of General Public Health, College of Public Health and Medical Science, Jimma University, Ethiopia; 2 Department of Reproductive Health and Human Nutrition, College of Public Health and Medical Science, Jimma University, Ethiopia; 3 Department of Epidemiology and Biostatistics, College of Public Health and Medical Science, Jimma University, Ethiopia

## Abstract

**Background:**

Good adherence to antiretroviral therapy is necessary to achieve the best virological response, lower the risk that drug resistance will develop, and reduce morbidity and mortality. Little is known about the rate and predictors of adherence in Ethiopia. Therefore this study determines the magnitude and predictors of adherence to antiretroviral therapy among people living with HIV/AIDS in Southwest Ethiopia.

**Methods:**

A cross sectional study was carried out from January 1, 2009 to March 3, 2009 among 319 adult PLWHA (≥ 18 years) attending ART clinic at Jimma university Specialized Hospital (JUSH). Multiple Logistic regression models were constructed with adherence and independent variables to identify the predictors.

**Results:**

About 303(95%) of the study subjects were adherent based on self report of missed doses (dose adherence) in a one-week recall before the actual interview. The rate of self reported adherence in the study based on the combined indicator of the dose, time and food adherence measurement was 72.4%. Patients who got family support were 2 times [2.12(1.25-3.59)] more likely to adhere than those who didn't get family support as an independent predictor of overall adherence (dose, time and food). The reasons given for missing drugs were 9(27.3%) running out of medication/drug, 7(21.2%) being away from home and 7(21.2%) being busy with other things.

**Conclusion:**

The adherence rate found in this study is similar to other resource limited setting and higher than the developed country. This study highlights emphasis should be given for income generating activities and social supports that helps to remember the patients for medication taking and management of opportunistic infections during the course of treatment.

## Background

The number of people living with HIV worldwide continued to grow in 2008, reaching an estimated 33.4 million [31.1 million-35.8 million]. Sub-Saharan Africa remains the region most heavily affected by HIV. In 2008, sub-Saharan Africa accounted for 67% of HIV infections worldwide, 68% of new HIV infections among adults. The region also accounted for 72% of the world's AIDS-related deaths in 2008 [1]. World Health Organization (WHO) recommendations on the use of antiretroviral therapy in resource-limited settings recognize the critical role of adherence in order to achieve clinical and programmatic success [2]. Good adherence to antiretroviral therapy is necessary to achieve the best virological response, lower the risk that drug resistance will develop, and reduce morbidity and mortality [3]. However, adherence barriers vary in different settings and lessons from more developed countries [4]. These benefits critically depend on patients achieving and maintaining high levels of medication adherence [5]. Very high levels of adherence (> 95%) are required for ART to be effective for long term and to prevent the emergence of resistant viral strains [6]. There has been a concern about the capability of patients in resource-limited settings to adhere to ART, especially in the African context [7].

Both clinical experience and emerging data suggest that many patients with chronic HIV disease do not fully adhere to their Highly Active Antiretroviral Therapy (HAART) regimens [8-11]. Incomplete adherence to antiretroviral agents can have serious consequences, including loss of plasma HIV suppression and turn lead to disease progression, inability to suppress HIV even with very intensive regimens, and development of drug resistant HIV strains. This can in transmission of resistant HIV to others [12-17].

However, introducing ART to sub-Saharan Africa was a topic of hot debate just a few years ago. Concerns about adherence and subsequent development of drug resistance, poor infrastructure, logistic and human capacity, and cost-effectiveness were the major issues [18]. In Ethiopia, the antiretroviral treatment program started with a fee-based ART program in 2003 then decentralized and free ART program in the Country was lunched since 2005[19]. Consequently, non-adherence to the proposed antiretroviral regimen is considered to be one of the greatest dangers to the response to treatment on an individual level and the dissemination of resistant viruses on the community level [20]. Little is known about the rate and predictors of adherence in Ethiopia. Therefore, this study determines the magnitude and predictors of adherence to antiretroviral therapy among people living with HIV/AIDS in Southwest Ethiopia.

## Methods

### Study setting

The study was conducted in Jimma University Specialized Hospital (JUSH). It is the only specialized referral Hospital in Southwest Ethiopia. Currently, it is giving service to more than 15,000,000 people living in Southwest Ethiopia. In 2002, the ART clinic of the hospital started its activity. After the government launched free ART in 2005, the hospital started to provide free service to People Living with HIV/AIDS (PLWHA). The study was conducted from January 1, 2009 to March 3, 2009 for a period of 2 month. The study design was a facility-based cross-sectional study. Institutional Ethical Review Committee of Jimma University approved the study and materials. All study subjects gave verbal informed consent.

### Participants

The source populations were all PLWHA on Highly Active Antiretroviral Treatment registered and following their treatment in Jimma University Specialized Hospital (JUSH). The study population for this study were adults who can fulfill the inclusion criteria- all PLWHA on HAART whose age is > 18 years regardless of their treatment category during the study period and available during data collection period. The exclusion criteria were: those patients on HAART whose age is < 18 years, adult (> 18 years) PLWHA who have been on treatment for less than 3 month period; potential participants at screening if they reported diabetes mellitus, current pregnancy. The sample size was calculated using Epi-info software version 6.04 StatCalc. Sample size was calculated using the 50% proportion (50% of respondent considered as adherence). A precision of 5% and with 95% confidence level was taken. A sample size was 290 which after adding 10% for non-response gave an overall sample size of 319. The study participants were selected randomly using a computer generated simple random table based on patient ART unique identification number.

### Measurement

The dependent variable was adherence to HAART among PLWHA. The independent variables were socio-economic status, socio-demographic factor, clinical characteristics, associated diseases and symptoms like diarrheal disease, anorexia, behavioral factors- alcohol intake, smoking habit, substance addiction. A structured pre-tested questionnaire which is developed from different literatures was used for data collection purpose. The questionnaire contains information on socio-demographic (age, sex, education, occupation, marital status), socio-economic variables(family income), psychosocial (social support, depression, active substance and alcohol use, disclosure of HIV serostatus, use of memory aids), disease characteristics (WHO clinical staging, duration of HIV infection), regimen related variables (dosing schedules and frequency, pill burden and complexity, dietary related demands, side effect, history of hospitalization), adherence to treatment information, symptoms associated with treatment. To identify clinical markers medical record was reviewed.

### Data analysis and processing

Data were edited, cleaned, coded and entered in to a computer and analyzed using SPSS- for windows version 16.0. Descriptive statistics was done to assess basic client characteristics. Bivariate analysis was done to determine presence of statistically significant association between explanatory variables and the outcome variable. All explanatory variables that were associated with the outcome variable in bivariate analyses were included in the final model. Multiple Logistic regression model was constructed with adherence and the independent variables to identify the predictors. The model was evaluated using forward stepwise selection method. Chi-square test and their p-values at the level of significance of 5% were used to define statistical associations between variables. Odds Ratios (OR) and their 95% CI were used to look into the strength of association between the dependent and independent variables. A person was said to be food adherent if he/she always followed dietary instructions agreed upon with the providers, otherwise he/she was labeled as non-adherent (Self-reported food adherence). Self-reported time adherence- where a person is said to be time adherent when claiming to always follow scheduling instructions otherwise non-adherent. Patients' self-report of whether any antiretroviral medication had been skipped on the day of interview, the previous day, the previous three days and the previous seven days before the interview was used to assess adherence to HAART. A person is said to be dose adherent when he/she took ≥ 95% of the prescribed doses correctly otherwise non-adherent (Self-reported dose adherence). Hence, for comparison purposes a combined indicator of adherence was made using the three adherence measures taking into account all questions pertaining adherence. So in this study Adherent is defined as when a PLWHA takes more than 95% (not missing a single doses of ART) of prescribed drug (dose adherence), follows time restriction (time adherence) and dietary instruction from health care provider (food adherence) for one week prior to the study otherwise Non-Adherent. This type of measurement of adherence has been used in similar setting and adherence in the previous seven days was used for comparison [21]. To assure quality of the data, the questionnaire was pre tested on PLWHA (5% of the sample size i.e. 21 individuals) and modifications were incorporated to the questionnaire and not included in the actual study. The interview was conducted in private room to create an atmosphere of empathy and confidence within a secure environment. An intensive 2 days training was given for all supervisors and data collectors before the process of data collection. The overall activity was controlled by the principal investigator of the study and proper designing of the data collection materials and continues supervision during data collection was performed. All completed questionnaire was examined for completeness and consistency during data management, storage and analysis.

## Results

### Socio demographic and economic characteristics

A total of 319 adult PLWHA participated in the study giving a response rate of 100%. Out of 319 PLWHA the largest number of participants, 148(46.4%) were in the age group 25-34 years and the mean (SD) age of the respondents was 35.08(7.73) ranging from 19 to 64 years and female constitutes 175(54.9%). The majority of the respondents 271(85.0%) were from Jimma City, 142(44.5%) participants were Oromo by ethnicity, 162(50.8%) Orthodox by religion, 155(48.6%) were married. The majority of them had 162(50.8%) attended elementary school. One hundred twenty nine (40.4%) were employed in private or governmental organizations and 174(54.5%) live with their parents. The median monthly income of the participants and their family were 300.00 and 350.00 Ethiopian Birr respectively, while weekly median expenditure for different purposes and for food preparation and purchasing were 100.00 and 50.00 Ethiopian Birr, respectively. The socio-demographic and economic characteristics of participants are presented in Table 1.

**Table 1:** Socio-demographic and economic characteristics of the study participants, Jimma University Specialized Hospital (JUSH), Southwest Ethiopia, 2009.

Characteristics	Frequency(Percentage)
Sex	

Male	144(45.1)

Female	175(54.9)

Age	

18-24	15(4.7)

25-34	148(46.4)

35-44	115(36.1)

≥ 45	41(12.9)

Permanent address	

Jimma	271(85.0)

Out of Jimma	48(15.0)

Ethnicity (N = 319)	

Oromo	142(44.5)

Amhra	84(26.3)

Dawro	40(12.5)

Kefa	24(7.5)

Gurage	15(4.7)

Others*	14(4.4)

Marital Status	

Married	155(48.6)

Single	67(21.0)

Windowed	43(13.5)

Divorced/Separated	54(16.9)

Educational status	

Illiterate	32(10.0)

Elementary	162(50.8)

Secondary	90(28.2)

12+	35(11.0)

Occupation	

Employed	129(40.4)

Merchant	31(9.7)

House Wife	42(13.2)

Daily laborer	84(26.3)

Have no job	20(6.3)

Others ***	13(4.1)

Living With	

Alone	77(24.1)

Family	54(16.9)

Parents	174(54.5)

Other	14(4.4)

Average Monthly income (N = 267)	

≤ 500	200(74.9)

501-999	26(9.7)

≥ 1000	41(15.4)

Religion	

Orthodox	162(50.8)

Muslim	78(24.5)

Protestant	68 (21.3)

Others**	11(3.4)

Average Family Income (N = 306)#	

≤ 500	216(70.6)

501-999	33(10.8)

≥ 1000	57(18.6)

*Tigre, Yem, Wolayita, Kenbata, Sidama, Bench,

**Catholic, Jova whiteness,

*** Farmer, Bar,

# Exchange rate 1 USD = 13 Ethiopian Birr (ETB)

### Clinical characteristics of the participants

Based on the review of patients' records, most of the participants 229(71.8%) were currently taking HAART with a regimen of Stavudine (d4T), Lamivudine (3TC) and Nivirapine (NVP) combination. Most of the participants 173(54.2%) started treatment in stage III of WHO disease classification and 217(71.1%) had a CD4 count of ≤ 200 cells/mm3 at the start of treatment and 155(60.3%) of the participants had recent CD4 count 201-499 cells/mm^3^; the range being 3 to 500 cells/mm3 and 42 to 1,230 cells/mm^3 ^with median of 144 cells/mm3 and 340 cells/mm3 at the beginning of treatment and recently at the time of data collection, respectively. Those who did not have CD4 count at the initiation of treatment had a median total lymphocyte count (TLC) of 1,000 cell/mm^3 ^with ranges from 700 cell/mm^3 ^to 2,103 cell/mm^3^. The majority of respondents 191(59.9%) received HAART for a duration of greater than 24 months with a mean duration of 26 months (Table 2).

**Table 2:** Clinical markers of the study participants comparing male and female using a Chi Square test, JUSH, South West Ethiopia, 2009

Characteristics	Male No. (%)	Female No. (%)	Total No. (%)	P - value
WHO disease stage when HAART started (N = 319)				0.075

I	7(4.9)	5(2.9)	12(3.8)	

II	35(24.3)	43(24.6)	78(24.5)	

III	85(59.0)	88(50.3)	173(54.2)	

IV	17(11.8)	39(22.3)	56(17.6)	

CD4 count when the treatment was started (N = 305)				0.298

≥ 500	1(0.7)	0(0.0)	1(0.3)	

201-499	43(30.5)	44(26.8)	87(28.5)	

≤ 200	97(68.8)	120(73.2)	217(71.1)	

Recent CD4 count (N = 257)				0.111

≥ 500	22(18.5)	38(27.5)	60(23.3)	

201-499	73(61.3)	82(59.4)	155(60.3)	

≤ 200	24(20.2)	18(13.1)	42(16.3)	

Duration of treatment in months (N = 319)				0.877

3.0-12.0	25(17.4)	28(16.0)	53(16.6)	

12.1-24.0	35(24.3)	40(22.9)	75(23.5)	

≥ 24.1	84(58.3)	107(61.1)	191(59.9)	

Treatment regimen(N = 319)				0.549

d4t (30)- 3TC-NVP	88(61.1)	115(65.7)	203(63.6)	

d4t (40)- 3TC-NVP	12(8.3)	14(8.0)	26(8.2)	

d4t (30)- 3TC-EFV	21(14.6)	17(9.7)	38(11.9)	

d4t (40)- 3TC-EFV	5(3.5)	3(1.7)	8(2.5)	

AZT-3TC-NVP	16(11.1)	25(14.3)	41(12.9)	

AZT-3TC-EFV	2(1.4)	1(0.6)	3(0.9)	

### Disclosure status, psychosocial support and behavioral factors of the participants

Majority of the respondents 290(90.9%) disclosed their HIV results to at least one person. One Hundred fifty (51.7%) of the respondents disclosed to their friends and 147(50.7%) to their wife or husbands, respectively. More females disclosed than males (56.6% Vs 43.4%). Majority of the respondents 265 (83.1) get family support (Table 3). The majority of the respondents did not take any substance; only 23(7.2%) of the participants take at least one type of substance (smoking, taking alcohol, chewing khat or other drugs). Out of these 10(3.1%) of them drunk alcohol, 17(5.3%) chewed khat and majority of them take those substance occasionally.

**Table 3:** Disclosure status and types of family support of the study participants, JUSH, southwest Ethiopia, 2009.

Characteristics	Frequency(Percentage)
Disclosure Status(HIV/AIDS) (N = 290) *	

Wife/husband	147(50.7)

Parents	129(44.5)

Children	81(27.9)

Neighbors	127(43.8)

Friends	150(51.7)

Relatives	99(34.1)

All relatives and Neighbors	53(18.3)

Others	6(2.1)

Support From family (N = 265)	

Emotional/Psychological	122(46.0)

Financial	41(15.5)

Physical care and support	63(23.8)

Food provision	39(14.7)

* More than one answer is possible.

### Rates of adherence and reasons for non adherence

**Figure 1:**
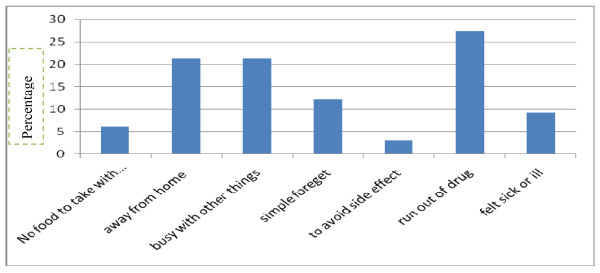
**Reasons given for missing to take ART medication among the respondents JUSH, South west Ethiopia, 2009**.

The three adherence measurements were assessed in the study to get a combined adherence indicator (Table 4). These include self reported missed doses, self reported schedule/time adherence and self reported food adherence. Accordingly, 303(95%) of the study subjects were adherent based on self report of missed doses (dose adherence) in a one-week recall. Two hundred fifty five (79.9%) of the study subjects always follow the schedule/time restrictions (time adherence) agreed upon with their providers and 286(89.7%) follow dietary instruction (Food Adherence). Hence, the rate of self reported adherence in the study area based on the combined indicator of the dose, time and food adherence measurement was 231(72.4%). The reasons given for missing drugs were running out of medication/drug 9(27.3%), being away from home 7(21.2%) and being busy with other things 7(21.2%) and the rest reasons included simply forgetting, having no food to take with the medication, fear of side effect and feeling sick or ill at that time (Figure 1).

**Table 4:** Self reported dose/treatment, Schedule/Program and food Adherence among the respondents JUSH, South west Ethiopia, 2009.

Characteristics	Frequency(Percentage)
Self Reported Dose Adherence (Last 7 Days) (N = 319)	

Adhered	303(95.0)

Not Adhered	16(5.0)

Self Reported Schedule Adherence (Last 7 Days) (N = 319)	

Adhered	255(79.9)

Not Adhered	64(20.1)

Self Reported Food Adherence (Last 7 Days) (N = 319)	

Adhered	286(89.7)

Non Adhered	33(10.3)

Over all Adherence (N = 319)	

Adhered	231(72.4)

Not Adhered	88(27.6)

### Predictors of adherence to HAART

The association of overall adherence (dose, time and food) with different variables were examined using binary logistic regression and there is a significant association (p < 0.05) identified with WHO stage, average family income, getting family support and sex and adherence of ART. Patients who got family support were 2 times [2.12(1.25-3.59)] more likely to adhere than those who didn't get family support as an independent predictor of overall adherence (dose, time and food) (Table 5).

**Table 5:** Final logistic regression model that predict adherence to dose, time and food in JUSH, Southwest Ethiopia, 2009.

Variables	Adherence		Crude OR (95% CI)	P-value	Adjusted OR (95% CI)	P-value
					
	Adhered N (%)	Non Adhered N (%)				
WHO stage				0.01		0.13

I	4(33.3%)	8(66.7%)	0.26(0.07-0.96)		0.17(0.042-1.18)	

II	56(71.8%)	22(28.2%)	1.31(0.62-2.74)		1.19(0.54- 2.56)	

III	134(77.5%)	39(22.5%)	1.76(0.91-3.41)		1.35(0.67- 2.72)	

IV	37(66.1%)	19(33.9%)	1.00		1.00	

Average family income Tertile				0.03		0.10

Lowest	64(69.6%)	28(30.4%)	1.00		1.00	

Middle	88(71.0%)	36(29.0%)	1.04(0.20-0.93)		1.07(0.58-1.98)	

Highest	70(77.8%)	20(22.2%)	1.53(0.23-0.98)		1.60(0.80-3.20)	

Getting family support				0.01		0.01

No	147(78.6%)	40(21.4%)	1.00		1.00	

Yes	84(63.6%)	48(36.4%)	0.48(0.29-0.78)		2.12(1.25-3.59)	

Sex				0.03		0.18

Male	113(78.5%)	31(21.5%)	1.76(1.06-2.93)		0.70(0.41-1.20)	

Female	118(67.4%)	57(32.6%)	1.00		1.00	

## Discussion

Antiretroviral therapy (ART) adherence levels of ≥ 95% optimize outcomes and minimize HIV drug resistance and to optimize measures of patient outcomes [22]. Previous studies in Ethiopia were using only self reported dose adherence as a measurement [23-25]. In our study we also used the time restriction (time adherence) and instructions related to food (food adherence) in addition to self reported dose adherence measurement. Our data suggest that adherence rates among patients in southwest Ethiopia were higher than adherence rates in most developed countries. In this study measuring adherence by patient self-report, 95% of the patients were adherent with ≥ 95% of prescribed doses in the last 7 days. Other studies conducted in developed countries demonstrated that the rates of adherence by self-report ranged from 40% to 70% [26-28]. Even in Botswana, fifty-four percent of patients in the study were adherent by self-report with 95% of prescribed doses [29]. Other studies in developing countries have shown comparable or better levels of individual adherence than what is seen in North American and European populations [29,30]. According to a prospective study in Southwest Ethiopia, 384 (96%) and 361(94.3%) of the study subjects were adherent based on self-report of missed doses (dose adherence) in a one-week recall at base line (M0) and follow up visit (M3) respectively. Three hundred eighty nine (97.2%) and 373 (97.4%) of the study subjects always followed the time restrictions (time adherence) agreed upon with their providers at M0 and M3 respectively. Three hundred thirty eight (84.5%) and 319 (83.3%) subjects followed instructions related to food (food adherence) all the time. Hence, the rate of self reported adherence in the study area based on the combined indicator of the three adherence errors was 79.3% at baseline and 75.7% at follow up visit [21]. Similarly, two studies in Ethiopia reported 81.2% and 82.8% adherence to more than 95% of doses [23,25]. This high rate of adherence showed adherence to ART in resource limited country can achieve a high level of adherence than those developed country. The overall rate of self reported adherence in this study based on the combined indicators of the three adherence errors was 72.4%. Similarly, consistent finding has been documented in similar set up [21]. Some studies in resource-rich settings have documented less than 50% of patients taking all their antiretroviral medications on time and according to dietary instructions [31,32]. Bonolo et al. review 43 articles on adherence to HAART. They found a mean rate of non-adherence of 30.4%, range from 5% to 67% [33]. This was much lower than our report confirming that patients in developing countries can achieve good adherence despite limited resources. The possible explanation for the greater adherence in our study might be the majority of the participants started ART recently, the participants were given strict adherence counseling sessions before starting ART in the hospital.

Non-adherence takes the form of skipping a dose. In a study of southwest Ethiopia, they found principal reasons reported for skipping doses were most 38 (43.7%) simply forget, 17 (19.5%) felt sick or ill at that time, and 11 (12.6%) ran out of medication at baseline. During the follow up visit again the majority 14 (65.6%) simply forgot, 4 (19%) felt sick and 4 (18%) were busy [21]. In our study the reasons given for missing drugs were running out of medication/drug 9(27.3%), being away from home 7(21.2%) and being busy with other things 7(21.2%) and the rest reasons included simply forgetting, having no food to take with the medication, fear of side effect and feeling sick or ill at that time. Forty-eight percent of patients asserted that they missed their doses due to finances, while 24% listed forgetting as a primary reason for treatment non-adherence. Other barriers to treatment included running out of medications (17%), travel/migration (13%), side effects (12%), and being too busy (12%) [29]. Forty-one percent of subjects (71/173) stated they never missed a dose of ARV. The 102 patients reporting missed doses at baseline did so for a variety of reasons, the most common of which was 'forgetting' to take the medication (41%; 42/102). Other reasons included being away from home (9%), being busy with other activities (6%), and taste perversion (5%), or concern about toxicity (4%). Less commonly listed reasons (2%) included running out of ARV medications or anxiety related to the constant reminder of their HIV infection [9]. Study subjects most commonly reported that they missed antiretroviral doses because they were busy or forgot, away from home, or experienced a break in their daily routine. Smaller proportions reported missing doses because they felt depressed or overwhelmed, were taking intentional drug holidays, or had run out of medication [34]. This implicate that the reason for skipping a dose should be given due emphasis from clinical, dispensing visit as well as during ongoing adherence counseling, and follow up visit. Other interventions aimed at maintaining adherence, and thereby optimizing the benefit of effective therapies should be sought in detail by health care workers.

There is good reason to expect that sociodemographic, psychosocial, and clinical variables should be associated with antiretroviral adherence and thus HIV disease activity [34]. In this study patients with average family income of middle and highest were more likely to have an overall adherence than the lowest average family income in bivariate analysis. The most common patient-related barriers were financial constraints [29,35]. Among patients having the economic ability to receive their medication, there was an association between the annual income and adherence [36,37]. Findings have also been inconsistent in defining the relationship of lower income [6,8,37,38] to adherence. A monthly middle income was significantly associated with greater pharmacy adherence. Low or high incomes groups showed a higher risk for pharmacy non-adherence/economic status, in particular patients with the highest monthly income when compared with monthly middle income, was retained as a predictor of poor adherence only in the best case scenario [39]. A recently published meta-analysis [40] examined the association between socio-economic status and adherence to antiretroviral therapy: out of 8 studies, only 2 prospective studies identified low income as a predictor of non-adherence. Other factors might be contributed for the difference between income and adherence like educational status. Other study also demonstrated that social support has a paramount important for adherence uptake. In our study patients who got family support were 2 times more likely to adhere than those who didn't get the family support. Another factor facilitated adherence was support from the family encouraging and helping to remind them to take the treatment. Social support, such as someone to help with the tasks of starting to rebuild a life, assistance with cooking and assistance to grow crops, all encouraged adherence [41]. Similarly, it has been reported in other studies [21] as social support was a constant predictor of adherence identified at baseline and follow up visit, living in a couple could improve adherence because it increases the routinization of daily behaviors and activities (Wagner & Ryan, 2004) [42] and better social supports for using medications were all associated with better adherence [34]. However, a recent meta-analysis of studies across multiple medical conditions determined that adherence was more strongly and consistently associated with functional support (i.e., practical/emotional support) than structural support (i.e., living arrangement/relationship status; DiMatteo, 2004) [43]. Within the domain of functional support, the study found that the provision of practical support had a significantly greater influence on adherence than emotional support [44]. Lacks of social support have been found to be associated with lower adherence [6,26]. Social support [36] was associated with greater adherence. Lack of support has been associated with an increase in suboptimal adherence [45,46]. Murphy and colleagues reported that those with greater social support for example having reassurance from family members, those having reliable alliances were more likely to be adherent over the past one month [47]. This highlights that social support assist in reminding to take the drugs according to the prescribed schedule and time, hence, for adherence. So it is better to advise/counsel our patients on initiation and continuation of HAART to be effective.

In our study disease stage/progression had been associated with adherence. Those participants who were in stage I were 74% less likely to adhere than those who are in the stage IV. Similar finding has been documented in other studies. In Chinese study, symptomatic disease stage had more likely to become adhere than asymptomatic disease stage [48]. Other factors significantly associated with viral suppression were less severe disease (WHO stage II or III vs WHO stage IV) [49]. Inconsistence to our finding in Cameroon, CDC stage B patients and specially CDC stage C patients had higher risk of pharmacy non-adherence than asymptomatic patients. When compared with asymptomatic patients, the multivariate analysis confirmed a marked risk of non-adherence for CDC stage B patients and CDC stage C patients in the worst-case scenario in Cameroon. However, HIV CDC clinical stage at the beginning of treatment significantly predicted loss to follow-up: compared with asymptomatic patients CDC stage A, CDC stage B patients and specially CDC stage C patients had greater rates of loss to follow-up [39].The possible reason might be those patients in stage I were not that much manifest the diseases/symptomatic and might feel that they are health looking as well not concerned about their illness as compared to those in advanced stage.

The findings of this study should be interpreted with some limitations. Because it was conducted at a single site, the findings may not be generalizable to dissimilar clinical settings. Recall bias and social desirability bias are also the possible bias which may encounter in this study. There is no gold standard for measuring adherence and our measurement of adherence is only based on patients' declarations of missed doses, scheduling instructions and dietary requirements. Despite the above limitations, the study addressed an important issue in developing country, and inclusion of several variables that predict adherence and to fully characterize the study population, we include other dimension of adherence measurement for successful treatment with ART (adhering to scheduling and to dietary instructions), reasonably large sample size (N = 319) and had a high participation rate.

## Conclusions

The adherence rate found in this study is similar to other resource limited setting and higher than the developed country. This study highlights emphasis should be given for income generating activities and social supports that helps to remember the patients for medication taking and management of opportunistic infections during the course of treatment. Further study should be carried out in longitudinal base as adherence is a dynamic behavioral and appropriate monitoring of patients' treatment apart from adherence is required to improve the treatment outcome. Identifying factors that contribute to non-adherence in large scale and site in follow up study should be given a due attention in the resource limited setting.

## Competing interests

The authors declare that they have no competing interests.

## Authors' contributions

AT conceived and designed the study, performed analysis and interpretation of data and drafted the manuscript, TB, FA and SB assisted with the design, interpretation of data and the critical review of the manuscript. All authors approved and read the final manuscript. All authors participated in critical appraisal and revision of the manuscript.
